# Cardiac Implications of Preeclampsia: A Review

**DOI:** 10.3390/jpm16050265

**Published:** 2026-05-15

**Authors:** Beani J. Forst, Linda R. Chambliss, David S. Majdalany

**Affiliations:** 1 Department of Cardiovascular Medicine, Mayo Clinic, Phoenix, AZ 85054, USA; 2 Department of Obstetrics and Gynecology, School of Medicine, Creighton University, Phoenix, AZ 85008, USA; lindachambliss@creighton.edu; 3 Department of Cardiovascular Medicine, Mayo Clinic, Rochester, MN 55905, USA

**Keywords:** preeclampsia, pregnancy-associated cardiovascular disease, peripartum cardiomyopathy, hypertensive disorders of pregnancy, chronic hypertension, endothelial dysfunction, personalized medicine

## Abstract

Preeclampsia (PE) is a multifactorial hypertensive disorder of pregnancy that significantly increases both short- and long-term cardiovascular risk for affected women. PE and cardiovascular disease (CVD) share common risk factors, including endothelial dysfunction, obesity, insulin resistance, and dyslipidemia. Women with a history of PE face a markedly elevated risk of chronic hypertension, heart failure, and adverse cardiac remodeling, with evidence suggestive of persistent vascular and myocardial changes after pregnancy. The complex pathophysiology of PE is multifactorial and is thought to involve a combination of abnormal placentation, immune dysregulation, and anti-angiogenic factors, which may induce permanent cardiovascular alterations. Genetic predispositions may further link PE with cardiomyopathies and peripartum cardiomyopathy. However, despite these well-established risks, standardized long-term surveillance and management strategies for women with prior PE remain lacking. Early identification and targeted intervention in women with a history of PE represent critical opportunities to mitigate future cardiovascular morbidity and mortality. This review highlights the urgent need for comprehensive, evidence-based strategies that incorporate personalized follow-up and risk stratification to improve cardiovascular outcomes in this high-risk population.

## 1. Introduction

PE is a disorder of pregnancy associated with new-onset hypertension (systolic blood pressure ≥ 140 mmHg and/or diastolic ≥ 90 mmHg), proteinuria or other specific end-organ dysfunction with onset after 20 weeks of gestation. It impacts 2% to 8% of pregnancies worldwide and is a leading cause of morbidity and mortality for pregnant women in the developed world [1]. Additionally, while fetal health can also be impacted, it carries both short- and long-term maternal cardiac complications [1].

**Figure 1:**
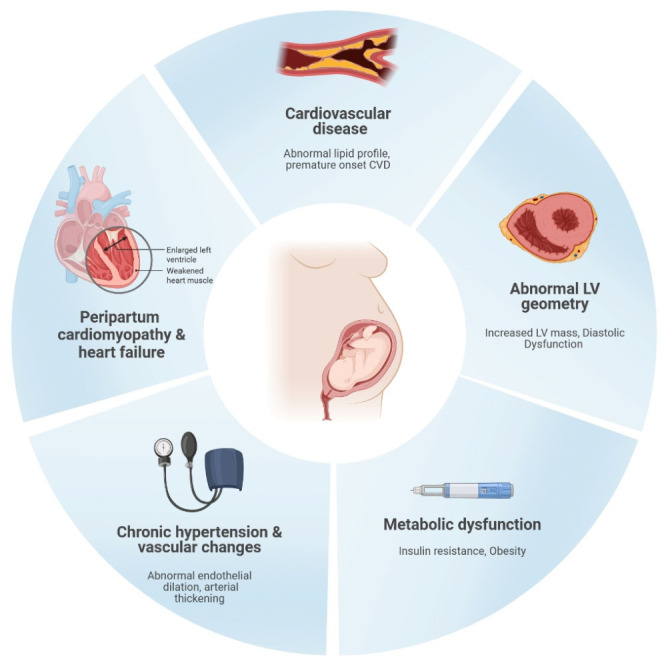
Long-term outcomes associated with preeclampsia. Image created in BioRender.

PE and CVD share many risk factors, including endothelial dysfunction, obesity, insulin resistance, diabetes mellitus, hypertension, and dyslipidemia (Figure 1). Meta-analyses have shown that PE is associated with a 4-fold increase in future incidence of heart failure and a 2-fold increased risk of coronary disease, stroke and death due to cardiovascular causes [2,3].

With the rising incidence of CVD among women under 55, there is a need to integrate and focus on female-specific conditions like PE, but its heterogeneity—ranging from mild to severe forms, with variable onset and associated comorbidities—necessitates individualized risk stratification and management. Genetic, metabolic, and biomarker profiling can help identify women at highest risk for both PE recurrence and future CVD, enabling tailored surveillance and intervention plans. This review aims to delineate the need for early diagnosis, intervention, and long-term monitoring to mitigate cardiovascular disease risks in this population.

## 2. Pathophysiology

The pathophysiology of PE is multifaceted and not well understood, but it has been thought to involve several interrelated processes primarily centered around abnormal placentation, immune system dysregulation and endothelial dysfunction [4]. A number of theories posit that with abnormal trophoblast invasion and subsequent incomplete spiral artery remodeling, a degree of placental ischemia develops, which then prompts the release of cytokines, anti-angiogenic markers and other downstream regulators that result in widespread endothelial dysfunction, vasoconstriction and systemic disease (Figure 1) [5,6].

There are two forms of PE: early-onset (<34 weeks gestation) and late-onset (≥34 weeks gestation), each hypothesized to have distinct pathophysiologic mechanisms. Early-onset PE has long been thought to reflect placental origins, while late-onset PE involves maternal factors, such as pre-existing hypertension or obesity; however, there are inconsistencies in both [7]. The placental origins hypothesis often lacks a causal connection, as many cases show normal placental histology and fetal growth, and common cardiovascular risk factors are frequently absent in individuals who develop PE later in gestation [8]. This magnifies that the relationship between cardiac function, placental implantation and PE development is complex, controversial and often resembles a “chicken vs. egg” scenario: does PE result in cardiovascular dysfunction or does a predisposition to cardiovascular dysfunction result in PE? The answer likely lies somewhere between the two.

**Figure 2:**
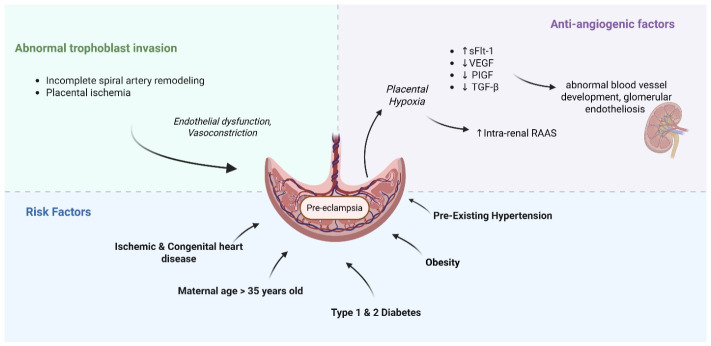
Proposed multifactorial pathogenesis of preeclampsia. Image created in BioRender.

Anti-angiogenic factors such as soluble FMS-like tyrosine kinase (sFLT-1) may play a significant role in the pathogenesis of PE by interfering with vascular endothelial growth factor (VEGF) and placental growth factor (PIGF), which impact blood vessel development and result in marked endothelial dysfunction (Figure 2) [9]. Maynard et al. determined that when pregnant and non-pregnant mice were injected with sFLT-1, high levels were sufficient to elicit hypertension and glomerular endotheliosis—a hallmark lesion of PE [10]. Pruthi et al. suggested that the cardiovascular changes induced by sFLT-1 are permanent and lay the foundation for future cardiovascular risk [11]. This hypothesis was recently supported by Garrido-Gimenez et al. in a prospective study of PE patients by cardiovascular risk assessment 12 years after the incident pregnancy [12]. Anti-angiogenic factors were measured in singleton pregnancies at the time of diagnosis of PE (both term and preterm) and were correlated over a decade later with various cardiac changes. Increased expression of sFlt-1 had a positive correlation with carotid intima-media thickness and left ventricular (LV) posterior wall thickness, while reduced PIGF levels correlated with reduced HDL, abnormal global longitudinal strain and elevated mean-arterial blood pressures (MAP) [12].

Women with underlying structural heart conditions, especially ischemic heart disease (IHD) and specific types of congenital heart disease (CHD), are more prone to chronic hypertension and consequently face an increased risk of developing hypertensive disorders during pregnancy [3]. A maternal age of 35 or older, obesity, and diabetes carry similar risk profiles [3]. Underlying cardiomyopathies and PE are also believed to share some similar pathophysiologic features, both demonstrating small vessel disease, endothelial damage and imbalanced angiogenic factors, as will be discussed later in this review [3]. Some studies have proposed that a genetic predisposition may underlie the association between PE and cardiomyopathy, with evidence suggesting that women who develop PE are more likely to harbor protein-altering mutations in genes implicated in cardiomyopathy [13].

The complex relationship between genetic predispositions, placental abnormalities, and cardiovascular factors emphasizes the heterogeneous nature of PE pathophysiology and underscores the need for further research to clarify these connections to enhance management strategies for affected women. In addition, by elucidating the roles of abnormal placentation, immune dysregulation, and endothelial dysfunction—along with the identification of key biomarkers such as sFLT-1 and PIGF—clinicians can better stratify risk and tailor monitoring for women with a history of PE.

## 3. Chronic Hypertension

PE, as has been described, is a hypertensive disorder unique to pregnancy that typically resolves within days to weeks of delivery as the maternal cardiovascular system returns to its pre-pregnancy state. However, chronic hypertension develops in approximately 30% of women within 10 years of the incident PE pregnancy [14,15]. Behrens et al. demonstrated that in the year following delivery, women who experienced PE during pregnancy had rates of hypertension 12 to 25 times higher than those with normotensive pregnancies. At 1 to 10 years postpartum, these rates remained 3 to 10 times higher, and even at 20 years postpartum, they remained twice as high [16]. This suggests that PE not only increases one’s short-term risk of CVD development but is a lifelong risk factor.

Although the pathophysiology predisposing women with PE to chronic hypertension is not fully understood, it is believed to be influenced by the aforementioned changes of PE that include acute vascular damage and atherosclerosis, endothelial dysfunction, inflammation, and oxidative stress [14,17]. Some studies suggest that these associated findings result in early vascular aging (EVA), defined by structural and biomechanical aortic changes resulting in a more rapid stiffening of the aortic wall, appearing years or even decades before typical vascular aging would occur [18,19]. Bokslag et al. conducted a prospective, observational study involving 187 women with a history of early-onset PE compared to those with uncomplicated pregnancies. They found that the rate of hypertension development in women with early-onset PE was similar to that of women who were 10 years older, indicating that these women have a cardiovascular profile akin to women a decade older [19]. Werlang et al. compared normotensive pregnancies against women with previous PE 6 months to 6 years postpartum (specifically those with severe, preterm and recurrent PE) and noted that even those already taking antihypertensive medications had increased brachial and central blood pressures, increased aortic stiffness, increased steady arterial load and increased arterial wave reflections [18]. Furthermore, women with PE with severe features had 7.87 times greater odds of EVA as compared with women without [18].

Shared risk factors for chronic hypertension such as elevated maternal BMI, diabetes and renal dysfunction provide an additive risk for the development of chronically elevated blood pressure (BP) in this cohort. Additionally, recurrence of PE (seen in approximately 15% of women) has been shown to carry an even higher risk ratio for chronic hypertension when compared to women with subsequent uncomplicated pregnancies [20].

Despite the well-documented predisposition to hypertension in these individuals, there remains a notable absence of standardized surveillance programs and structured follow-up protocols and guidelines for monitoring BP. Brown et al. propose that a lower upper limit of normal should be used for screening in the post-PE population. Their findings indicate that the upper limit of normal (mean + 2 SD) for women with normotensive pregnancies 6 months postpartum is 122/79 mmHg [21]. They argue that relying on traditional BP cutoffs results in a significant number of patients being overlooked, thereby extending their exposure to increased afterload in an already at-risk population [21]. Furthermore, the benefits of antihypertensive treatment have not been established from studies involving cohorts of young parous women who are often breastfeeding; instead, these benefits are typically derived from studies involving older cohorts, predominantly consisting of men. Benschop et al. emphasize the significance of using ambulatory blood pressure monitoring (ABPM) in this population, as they followed 200 women one year after experiencing PE with severe features, utilizing both ABPM and office blood pressure monitoring [22]. Of these women, 41.5% were diagnosed with hypertension; however, based on office BP measurement alone, only 24.5% of patients would have received the appropriate diagnosis and subsequent initiation of hypertensive treatment [22]. This study underscores the critical role that ABPM plays in this cohort, and its use should be closely examined in the postpartum and primary care setting. Developing tailored hypertension screening guidelines and management strategies with advanced monitoring techniques could facilitate early detection and initiation of appropriate treatment, ultimately improving long-term health outcomes for this at-risk population.

## 4. Left Ventricular Remodeling and Alterations in Cardiac Function

In a normal pregnancy, there is an increase in left ventricular (LV) muscle mass, ventricular wall thickness, and the diameters of the right atrium and ventricle [23]. This is accompanied by an increase in heart rate, stroke volume and cardiac output with decreased systemic vascular resistance supporting the heightened metabolic demands of pregnancy. Even with these changes, the LV contractile function remains preserved. If any alterations in cardiac geometry occur, they are normally reversible within three months of the postpartum period in normotensive women [23].

PE, however, is associated with after-load-mediated LV remodeling, concentric remodeling, eccentric hypertrophy and concentric hypertrophy, resulting from elevated total vascular resistance [24]. Both preterm and term PE reveal mild-to-moderate LV diastolic dysfunction and impaired myocardial relaxation, as well as elevated brain natriuretic peptide (BNP) levels [24,25]. However, early-onset PE is often associated with more severe cardiac impairment and greater long-term myocardial damage [25,26]. More specifically, studies have revealed that women with early-onset PE have persistent diastolic dysfunction at 6 months and one year postpartum when compared to late-onset PE and low-risk pregnancies [25,26,27,28,29]. In fact, Melchiorre et al. demonstrated that the persistence of asymptomatic moderate–severe LV dysfunction and hypertrophy at one year postpartum is as high as 56% in preterm PE patients compared to term PE (14%) and normal controls (8%) [24]. Only a few longitudinal studies currently exist beyond the one year postpartum period in PE cohorts, but they reveal elevated myocardial mass, abnormal strain, and left atrial enlargement between 2 and 12 years after the incident PE pregnancy [12,30]. Future studies are required to determine how the development and persistence of these postpartum subclinical cardiac abnormalities serve as markers of cardiovascular risk across a woman’s lifetime and how best to integrate screening.

Cardiac function has also been evaluated at the time of delivery in PE patients, revealing higher right ventricular (RV) systolic pressures, decreased global longitudinal systolic strain, elevated LV filling pressures and progressive diastolic dysfunction when compared to cohorts of healthy pregnant patients [31]. These hemodynamic and structural abnormalities contribute to the pathogenesis of pulmonary edema that can predispose to acute decompensation and become a potentially life-threatening complication of hypertensive crises in pregnancy that frequently necessitates preterm delivery, placing not only the mother but the neonate at risk.

Advanced echocardiographic techniques, including speckle-tracking for strain analysis and tissue Doppler imaging, can detect subtle myocardial dysfunction in women with PE, often before symptoms or a drop in ejection fraction [32]. Incorporating this into routine peri- and postpartum cardiovascular assessment in women with PE, particularly those with early-onset or severe disease, may facilitate timely intervention and future risk stratification. By identifying high-risk women who benefit from individualized follow-up, targeted prevention strategies can be developed to reduce long-term cardiovascular morbidity.

## 5. Peripartum Cardiomyopathy and Heart Failure

Peripartum cardiomyopathy (PPCM) is a rare but serious form of heart failure (HF) (LV ejection fraction < 45% or fractional shortening < 30% or both) that occurs during the last month of pregnancy or within five months after delivery without any other explanation. The development of PPCM in patients with a history of PE is particularly concerning as the conditions are characterized by similar recurrence and mortality rates in subsequent pregnancies [33]. Early recognition and prompt treatment are essential to optimize outcomes.

A meta-analysis by Bello et al. and the PPCM registry of the EURObservational Research Programme found that the prevalence of PE in women with PPCM is approximately 22%, which is over four times the global average [34,35].

The exact mechanisms linking PE to PPCM are not fully understood but likely involve a complex relationship between persistent endothelial dysfunction resulting in impaired vasodilation, increased vascular resistance, and underlying structural cardiac changes [36]. The imbalance of sFLT1 and PIGF, as previously discussed, plays an intricate role in the development of PE, but has also been associated with the development of dilated cardiomyopathy (DCM) [37].

Gumilar et al. explored the potential genetic predispositions for PE and PPCM, emphasizing that both conditions are linked to genetic irregularities affecting vascular function, immune response, thrombophilia, and BP regulation [36]. Through a literature review, they identified eight genes with overlapping roles in both conditions: CD274 (PD-L1), sFLT1, PIGF, PCDC1, VEGFA, SERPINE1, GNB3, and MIR146A [36]. Gammill et al. performed whole-exome sequencing of PE and PCCM patients and noted that 73% of PE patients had loss-of-function variants in the TTN gene, one of the most common causes of heritable DCM [13]. Identifying these genes and variants may offer insights into the shared pathophysiological mechanisms of PE and PPCM, potentially leading to targeted therapeutic strategies for managing both conditions and introducing the concept of genetic counseling for conditions previously thought not to have a genetic predisposition.

The association between PE and late-onset cardiomyopathy (CM) has not been as well characterized as PPCM; however, recent studies suggest that women with a history of PE have significantly higher rates of CM compared to those with normotensive pregnancies [38]. Specifically, PE with severe features has been associated with an adjusted hazard ratio (HR) of 2.20 and moderate PE with an HR of 1.89. These increased risks persisted for more than 5 years post-pregnancy [38]. Interestingly, a sub-analysis revealed that only 50% of those who developed CM had been diagnosed with chronic hypertension after pregnancy, suggesting that the elevated risk of HF may not primarily be due to the increased risk of PPCM or IHD typically linked with PE [38,39]. The Cardiovascular Disease in Norway Project found that those who developed PE during a single lifetime pregnancy were twice as likely to later develop heart failure over a median 11.8 years of follow-up, and those who developed PE in more than one pregnancy were four times as likely; however, no clear causal mechanism was identified [40].

Because of the greater LV wall thickness and ventricular mass associated with PE and other hypertensive disorders of pregnancy (HDPs), as well as many overlapping cardiometabolic risk factors, PE is hypothesized to represent a sex-specific risk factor for heart failure with preserved ejection fraction (HFpEF) [41]. Williams et al. showed that after adjusting for baseline hypertension and other covariates, women with PE with severe features early on were more likely to be hospitalized for HFpEF, with a median time from delivery to diagnosis of 32 months [42]. This introduces a newly recognized disease entity of peripartum HFpEF, suggesting that peripartum HF may encompass a spectrum of both systolic and diastolic dysfunction [43].

These findings highlight the importance of early recognition, long-term cardiovascular monitoring, and consideration of genetic counseling in women with a history of PE or PPCM, as both conditions may reflect a broader, sex-specific vulnerability to heart failure.

## 6. Gestational Lipid Levels, Acute Atherosis, Vascular Remodeling and Cardiovascular Disease

In a normal late gestational period, there is a 3-fold increase in circulating triglycerides (TG) and a 50% increase in total cholesterol (mostly presenting as LDLs) secondary to a combined stimulation of hormone-sensitive lipase by human placental lactogen (HPL), increased insulin resistance and increased hepatic output of VLDL by circulating estrogen [44,45]. At 6–10 weeks after delivery, these levels revert to those from pre-pregnancy. In PE, however, the lipid levels typically do not fully return to normal and while total levels remain similar to those in normotensive patients, there is excessive circulating maternal hypertriglyceridemia, free fatty acids (FFAs), increased concentration of small LDL and reduced high-density lipoprotein (HDL) [45,46]. This may explain why women with a history of PE show an increased susceptibility to lipoprotein oxidation compared to those with normal pregnancies [47,48].

Although triglycerides (TGs) are not directly atherogenic, they serve as an important biomarker for cardiovascular disease (CVD) risk due to their association with other atherogenic lipoproteins that promote atherogenesis independently of LDL [49]. Much like the mechanism in coronary arteries, TGs in the endothelial cells lining the uterine spiral arteries and radial arteries that supply the decidua and myometrium lead to decreased prostacyclin production and increased oxidative stress, promoting the release of free fatty acids (FFAs) and apolipoproteins, a process known as acute atherosis of the placenta [50]. These lesions are histologically similar to early-stage atherosclerosis, defined by subendothelial lipid-laden foam cells, fibrinoid necrosis of the arterial wall, and perivascular lymphocytic infiltration [51]. Adank et al. demonstrated that lipid levels in early pregnancy were positively associated with the prevalence of metabolic syndrome and lipid levels six years after pregnancy, even after correction for smoking and pre- and post-pregnancy BMI [52].

Acute atherosis is exceedingly rare in normal pregnancies but is seen in approximately half of PE pregnancies and has also been documented in pregnancies complicated by DM and gestational and chronic hypertension [53,54]. It is hypothesized that the narrowed vessel caliber of these placental arteries exacerbates placental dysfunction and oxidative stress, which in turn contributes to reduced placental perfusion and the release of inflammatory substances from the placenta into maternal circulation, which contributes to the development of PE [55]. Although acute atherosis has traditionally been considered a transient placental lesion that regresses after delivery, its histopathologic resemblance to systemic atherosclerosis—combined with its strong association with PE—supports a mechanistic link between PE and premature CVD. This is reinforced by imaging data from the CPH-PRECIOUS trial, which demonstrated a significantly higher prevalence of coronary atherosclerosis on coronary computed tomography angiography (CCTA) among women aged 40–55 years with prior PE, even after adjustment for dyslipidemia, DM, smoking, BMI, menopause, and parity [56]. Despite the number needed to screen to detect coronary atherosclerosis being only <4 in those ≥45 years of age and approximately 5 for those aged 40–<45 years, CCTA is still not used in current practice in this population unless traditional ischemic symptoms are present [56]. Furthermore, 16.6% of women with prior PE (mean age 46.8 years) had detectable coronary artery calcium (CAC), but 10.8% had exclusively noncalcified plaque—lesions that would have been missed with CCTA [56]. Carotid intima-media thickness (CIMT) ultrasound has thus been proposed as a modality for the detection of this early-stage subclinical atherosclerosis; however, research on the use of this screening modality in this population is exceptionally limited. One recent meta-analysis of over 20,000 women demonstrated an increased CIMT in PE cohorts; specifically, pooled analysis showed that the odds of having subclinical atherosclerosis were 1.57 times higher in women with histories of PE compared to controls [57].

Beyond imaging markers, epidemiologic data further support a shared cardiovascular predisposition between PE and CVD. Egeland et al. demonstrated that a family history of myocardial infarction (MI) before the age of 60 in a first-degree relative is associated with an increased risk of PE (odds ratio 1.8, 95% CI: 1.31–2.39) [58]. This evidence supports integrating family history of CVD into PE risk assessment and prevention and highlights the need to consider pre-existing cardiovascular risk profiles in these patients [59]. It also reinforces the importance of further research to define the genetic architecture underlying PE and to test targeted interventions.

Taken all together, these findings suggest that the vascular pathology underlying PE may not fully resolve postpartum but instead reflects—or initiates—an accelerated atherosclerotic trajectory. Despite this, there are no dedicated lipid-lowering targets or CVD screening paradigms for women with prior PE.

## 7. Proposed Screening Timeline

The limited availability of randomized controlled trials and the exclusion of pregnant women from trials create uncertainty among clinicians, who must rely on observational data and extrapolation from general CVD guidelines, often leading to inconsistent management, underscreening, and undertreatment. This contributes to missed opportunities to identify and treat modifiable risk factors early in a high-risk population. To help address this, we propose a pragmatic, risk-stratified surveillance timeline informed by the literature reviewed here and the current American College of Obstetrics and Gynecology, American Heart Association/American College of Cardiology, and European Society of Cardiology recommendations (Table 1 and Table 2).

Blood pressure should be reassessed with an in-person evaluation within 7–10 days of delivery in all women with any hypertensive disorder of pregnancy [60,61]. Home blood pressure monitoring should be performed from discharge until the first postpartum visit to facilitate early detection of persistent or worsening hypertension and to guide timely medication adjustment. Repeat assessments should be performed at 12 weeks postpartum to determine if there has been a transition to chronic disease requiring formal diagnosis and further treatment evaluation [62]. Ambulatory blood pressure monitoring should remain an option for any patient in whom white coat or masked hypertension is suspected. Clear transition of care to a primary care clinician or cardiologist with explicit documentation of PE is essential to ensure sustained longitudinal blood pressure surveillance.

Fasting lipid panel and glycemic screening (fasting glucose or HbA1c) should be performed within 3–6 months postpartum, with transition to at least annual monitoring thereafter if findings are within normal limits [63].

For long-term atherosclerotic risk assessment, consideration of coronary artery calcium (CAC) scoring beginning at approximately age 40–45 years—or earlier in those with additional risk enhancers—may help identify accelerated vascular aging not captured by traditional risk calculators [64]. These women are frequently risk-underestimated by pooled cohort equations, and CAC can be particularly useful for risk reclassification and therapeutic decision-making. Postpartum cardiovascular surveillance should include an initial lipid panel within the first year after delivery, typically 3–12 months once pregnancy-related metabolic changes have normalized. If results are within normal limits, repeat screening every five years is reasonable, consistent with general primary prevention guidance, with earlier or more frequent testing if additional risk factors such as hypertension, obesity, or gestational diabetes emerge. Although specific LDL-C targets have not been established for women with prior PE, most experts apply an enhanced primary prevention framework. Statin therapy can be reasonably considered for LDL-C ≥ 160 mg/dL in the presence of this female-specific risk enhancer, with shared decision-making for LDL-C levels of 130–159 mg/dL, particularly when additional risk factors or subclinical atherosclerosis are present [65]. LDL-C ≥ 190 mg/dL should be treated according to standard severe hypercholesterolemia guidelines regardless of pregnancy history [65]. Given the paucity of prospective, randomized data in this specific cohort, dedicated lipid studies are needed to define optimal LDL-C targets, timing of initiation, and long-term outcomes.

At least one baseline transthoracic echocardiogram (TTE) should be performed within 10 years of the index pregnancy in all women with prior PE, regardless of severity, given the risk of subclinical LV remodeling and diastolic dysfunction [42,66]. However, in those with a history of PE with severe features, persistent hypertension, cardiopulmonary symptoms and abnormal exam findings, the first echocardiogram should be performed during the peripartum or early postpartum period [42]. Subsequent imaging and follow-up should be individualized based on echocardiographic findings, clinical trajectory, and overall CVD risk profile, reflecting a personalized approach to long-term surveillance.

**Table 1:** Summary of long-term cardiovascular outcomes associated with prior preeclampsia.

Cardiovascular Outcome	Relative Risk (RR)/Hazard Ratio (HR)	Timeframe	Modifying Factors	References
Chronic Hypertension	RR 3.13 (95% CI 2.51–3.89) • HR 18.5 (95% CI 16.52–20.78) in first year • HR 4.9 (95% CI 4.64–5.22) at 5–10 years	Within 10 years (≈20% develop within 15 years). Highest risk in first year; persists lifelong.	Early-onset PE, recurrent PE, PE severity	[67,68]
Heart Failure	HR 2.0 (95% CI 1.50–2.68) • RR 4.19 (95% CI 2.09–8.38)	<1 year through 10 years postpartum	Recurrence; early-onset PE	[2,40]
Cardiovascular Disease (ASCVD)	HR 1.72 (95% CI 1.42–2.10) • RR 2.50 (95% CI 1.43–4.37)	Earlier onset vs. women without PE	Severity, recurrence, early-onset PE	[2,56,69]
Metabolic Syndrome	RR 1.49 (95% CI 1.27–1.75) to 3.01 (95% CI 1.85–5.71)	6 months–7 years postpartum	Early-onset PE; gestational diabetes	[63,70]
LV Diastolic Dysfunction	HR 2.09 (95% CI 1.80–2.44)	Mean 32.2 months postpartum	Early-onset PE	[42]

**Table 2:** Structured surveillance plan and clinical timeline follow-up after preeclampsia.

Timepoint	Test/Action	Setting	Purpose
Discharge to 7–10 days postpartum	Home BP monitoring (daily or as clinically indicated)	Patient at home; OB/MFM/PCP reviews	Early detection of persistent/worsening HTN; guide medication titration.
7–10 days PP	In-person BP assessment	OB/MFM/PCP	Required for all with any HDP; assess control, symptoms, meds. Consider labs if indicated.
Any time PP as indicated	Ambulatory BP Monitoring (ABPM)	OB/MFM/PCP/Cardiology	Use if white coat or masked HTN suspected to refine diagnosis/management.
12 weeks PP	BP reassessment	OB/MFM/PCP	Determine transition to chronic hypertension if BP remains elevated. Set long-term plan.
3–12 months PP	Fasting lipid panel	PCP/Cardiology	Baseline ASCVD risk factors after pregnancy. If normal, transition to 5-year monitoring (more frequent if additional risk factors).
3–6 months PP	Glycemic screening (fasting glucose or HbA1c)	PCP	Screen for DM. If absent, transition to annual monitoring.
Annual Follow-Up	BP check; lipid panel & glycemic screening if prior results normal	PCP/Cardiology	Ongoing surveillance for HTN, dyslipidemia, diabetes, and global CVD risk.
Peripartum/early PP	Echocardiogram if PE with severe features	PCP/Cardiology	Early imaging for those at higher cardiac risk; guides earlier intervention.
At least once within 10 years of index pregnancy	Echocardiogram	PCP/Cardiology	Screen for subclinical LV remodeling/diastolic dysfunction even if asymptomatic.
Age 40–45 (earlier if additional risk enhancers)	Coronary Artery Calcium (CAC) score	PCP/Cardiology	Consider detecting accelerated vascular aging not captured by traditional risk calculators; may inform statin decisions.

## 8. Conclusions

PE is a complex disorder with significant implications for maternal health that extend well beyond pregnancy into long-term cardiovascular risks. The intricate interplay between PE and CVD is underscored by their many shared risk factors, such as endothelial dysfunction, hypertension, dyslipidemia, and obesity, as well as potential genetic predispositions. The development of chronic hypertension, heart failure, and alterations in cardiac function in association with PE highlights the need for comprehensive monitoring and management strategies. Currently, guidelines concerning cardiovascular health in women mention the increased risk burden incurred by a hypertensive pregnancy disorder; however, well-founded, evidence-based recommendations are lacking regarding screening, intervals, and prevention. Importantly, the occurrence of PE in otherwise young women provides a critical and time-sensitive opportunity for early risk identification. Unlike many traditional cardiovascular risk factors that emerge later in life, PE may serve as an early sentinel event—revealing latent vascular dysfunction decades before overt clinical disease develops. This creates a unique window for the timely implementation of preventive strategies, including aggressive risk factor modification, structured blood pressure monitoring, metabolic screening, and targeted imaging when appropriate. Such early intervention has the potential to meaningfully reduce not only individual lifetime morbidity but also the broader societal and economic burden of cardiovascular disease in women. As research continues to elucidate the molecular, genetic, and hemodynamic mechanisms linking PE and CVD, it is imperative that emerging insights be translated into clinical practice. This evolving understanding offers a powerful avenue to advance personalized medicine—facilitating refined risk stratification, tailored surveillance strategies, and individualized prevention plans that reflect each patient’s unique genetic, molecular, and clinical profile. Integrating pregnancy history into routine cardiovascular care represents a paradigm shift in women’s health, transforming PE from an isolated obstetric complication into a pivotal component of lifelong cardiovascular risk assessment and prevention.

## Data Availability

No new data were created or analyzed in this study. Data sharing is not applicable to this article.
